# Thyroid Hormone Understanding Branches Out: Insights into PBDE Impacts on Brain Development

**DOI:** 10.1289/ehp.119-a80a

**Published:** 2011-02

**Authors:** Kellyn S. Betts

**Affiliations:** **Kellyn S. Betts** has written about environmental contaminants, hazards, and technology for solving environmental problems for publications including *EHP* and *Environmental Science & Technology* for more than a dozen years

Polybrominated diphenyl ether (PBDE) flame retardants and their hydroxylated metabolites are structurally similar to thyroid hormone. A detailed study now provides one of the most insightful assessments to date of how environmentally relevant levels of PBDEs may impair brain development by interfering with thyroid hormone receptor (TR)–mediated transcription **[*EHP*** **119(2):168–175; Ibhazehiebo et al.]**.

PBDEs were used for decades as flame retardants in a wide variety of consumer and household goods, and U.S. citizens are widely exposed to them. Scientists know that PBDEs can cross the blood–brain barrier and accumulate in the central nervous system, and a growing body of evidence implicates these chemicals as developmental neurotoxicants. PBDEs and their metabolites have been detected in fetal blood, liver, and placenta, and in human milk.

Thyroid hormone deficiency in the perinatal period can cause abnormal brain development. The study authors studied how PBDEs affected the mechanisms underlying thyroid hormone action in the developing brain using an established rodent cerebellum model. The experiments were designed to distinguish between effects on TR-mediated gene transcription due to 1) altered interactions between thyroid hormone and the TR and 2) altered interactions between the TR and short DNA sequences known as thyroid hormone response elements (TREs).

The scientists initially expected to demonstrate that PBDEs displaced thyroid hormone from the TR site. Instead they found PBDEs may prevent the TR from interacting with TREs, possibly through effects on the TR DNA-binding domain that normally binds to TREs in thyroid hormone–regulated genes.

The team identified two PBDE compounds, BDE-209 and BDE-100, as playing important roles in suppressing transcription from several TREs. None of the hydroxylated PBDE metabolites evaluated significantly suppressed TR-mediated transcription.

Decreases in thyroid hormone levels have been shown to alter the complex treelike branching of Purkinje cell dendrites, which is critical to normal brain development. The authors previously reported effects of polychlorinated biphenyls on dendrites that they determined were mediated by effects on the TR. The current study showed the action of BDE-209 on TR-mediated transcription also inhibited the growth and branching, or arborization, of Purkinje cell dendrites. These effects may disrupt other aspects of brain development, given that TR-mediated gene expression occurs in many other cells.

The scientists say their work also suggests the effects they observed are associated with additional pathways, such as disruption of intracellular signaling pathways that rely on calcium ion homeostasis. It is unknown how many genes have TREs; therefore, the new work indicates an important next step will be to unearth the mechanism by which PBDEs can disrupt or suppress those TRE-mediated development genes.

